# A Small-Molecule Fluorescent Probe for the Detection of Mitochondrial Peroxynitrite

**DOI:** 10.3390/molecules28247976

**Published:** 2023-12-06

**Authors:** Han Dong, Meng-Yu Tang, Shili Shen, Xiao-Qun Cao, Xiao-Fan Zhang

**Affiliations:** Institute of Optical Functional Materials for Biomedical Imaging, School of Chemistry and Pharmaceutical Engineering, Shandong First Medical University & Shandong Academy of Medical Sciences, Taian 271016, Chinaxqcao@sdfmu.edu.cn (X.-Q.C.)

**Keywords:** fluorescent probe, peroxynitrite, mitochondria, cellular imaging

## Abstract

Reactive oxygen species (ROS) are pivotal signaling molecules that control a variety of physiological functions. As a member of the ROS family, peroxynitrite (ONOO^−^) possesses strong oxidation and nitrification abilities. Abnormally elevated levels of ONOO^−^ can lead to cellular oxidative stress, which may cause several diseases. In this work, based on the rhodamine fluorophore, we designed and synthesized a novel small-molecule fluorescent probe (**DH-1**) for ONOO^−^. Upon reaction with ONOO^−^, **DH-1** exhibited a significant fluorescence signal enhancement (approximately 34-fold). Moreover, **DH-1** showed an excellent mitochondria-targeting capability. Confocal fluorescence imaging validated its ability to detect ONOO^−^ changes in HeLa and RAW264.7 cells. Notably, we observed the ONOO^−^ generation during the ferroptosis process by taking advantage of the probe. **DH-1** displayed good biocompatibility, facile synthesis, and high selectivity, and may have potential applications in the study of ONOO^−^-associated diseases in biosystems.

## 1. Introduction

Reactive oxygen species (ROS) and reactive nitrogen species (RNS), e.g., peroxynitrite (ONOO^−^), nitric oxide (NO), hypochlorous acid (HOCl), and superoxide anion (O_2_^•−^), are a class of substances with extremely high reactivity [1,2,3]. Among them, ONOO^−^, which is formed through the reaction of NO with O_2_^•−^, has a low concentration and short life span [4,5]. On the one hand, the high reactivity of ONOO^−^ is due to its intrinsic strong oxidizing and nitrifying abilities, which can directly react with the target molecules. On the other hand, ONOO^−^ can be converted to secondary radical species, such as decomposition to •OH, or reaction with CO_2_ to produce CO_3_^•−^, which then reacts with the target molecules [6,7,8]. Therefore, ONOO^−^ can destroy many important biomolecules (such as nucleic acids, proteins, and lipids) [9,10,11,12,13]. These biochemical properties make ONOO^−^ an important predisposing factor for many diseases, including cardiovascular diseases, neurodegenerative diseases, inflammatory diseases, and cancer [14,15,16,17,18,19]. However, the high reactivity of ONOO^−^ also plays a critical role in cell signaling processes, such as the nitrification of key proteins to regulate signaling pathways. In addition, ONOO^−^ has an antibacterial effect and can be produced in large quantities during the process of deploying macrophages to fight against invading pathogens, such as bacteria, thereby helping to kill such pathogens. Hence, monitoring the ONOO^−^ changes in biosystems is highly important.

Currently, small-molecule fluorescent probes have gained widespread interest and have become an effective means of detecting specific analytes, due to their high sensitivity, rapid response ability, unparalleled selectivity, and real-time measurement capability [20,21,22,23,24,25,26,27,28,29,30,31,32,33,34,35]. In addition, fluorescent probes, combined with cellular confocal imaging, have been useful tools for elucidating the biological functions of the analyte of interest. Among the commonly used fluorophores (e.g., coumarin, naphthalimide, BODIPY, cyanine), rhodamine dyes possess the advantages of good photostability, improved signal-to-background ratio, easy preparation, and high fluorescence brightness [36,37,38,39]. Nowadays, many rhodamine-based derivatives have been developed for the in vivo specific measurement of ONOO^−^ [40,41,42,43,44].

Herein, we developed a new ONOO^−^-responsive molecular fluorescence probe (**DH-1**) by introducing a ferrocene moiety into the rhodamine framework. The probe itself is non-fluorescent; upon reaction with ONOO^−^, a remarkable fluorescence increase in the probe is observed in the in vitro tests and the fluorescence intensity shows good linearity. **DH-1** exhibits higher selectivity and responsiveness towards ONOO^−^ compared to other ROS/RNS. Notably, **DH-1** can specifically target mitochondria with good biocompatibility. In addition, **DH-1** has been successfully utilized for the fluorescence confocal imaging of ONOO^−^ after exogenous and endogenous drug stimulations in living cells. More importantly, we employed **DH-1** to detect ONOO^−^ fluctuations in the ferroptosis process.

## 2. Results and Discussion

### 2.1. Design and Synthesis of the Probe ***DH-1***

**Scheme 1:**
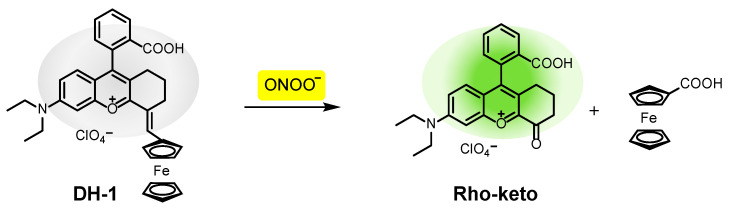
Mechanism of the probe **DH-1** for sensing ONOO^−^.

The probe **DH-1** was designed based on a rhodamine fluorophore. The introduction of a ferrocene unit into the probe structure could quench the fluorescence of **DH-1**, and no fluorescence emission was noted. By contrast, after reaction with ONOO^−^, the C=C bond of **DH-1** was oxidized and cleaved to form **Rho-keto**, which emitted a significant fluorescence signal (Scheme 1), thereby realizing the detection of ONOO^−^.

**Scheme 2:**
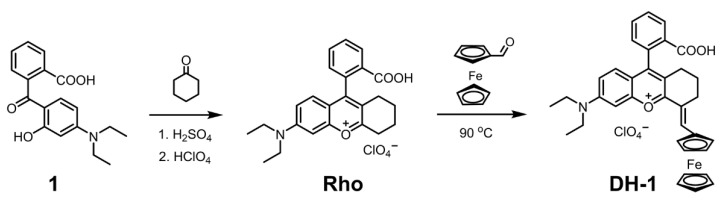
Synthesis of the probe **DH-1**.

**DH-1** was synthesized in two steps, as shown in Scheme 2. First, cyclohexanone was condensed with compound **1** to gain intermediate **Rho**. Next, **DH-1** was obtained through the aldol condensation reaction between **Rho** and ferrocenecarboxaldehyde with a 79% yield. It is worth noting that the alpha carbon of the carbonyl group is highly reactive, so ferrocene aldehyde is added at exactly the position of the alpha carbon in the cyclohexane fragment of rhodamine (Scheme 2). **DH-1** was characterized using NMR spectra and HRMS. The characterization data are provided in the Supporting Information (Figures S1–S3).

### 2.2. Spectral Response of the Probe ***DH-1*** to ONOO^−^

**Figure 1:**
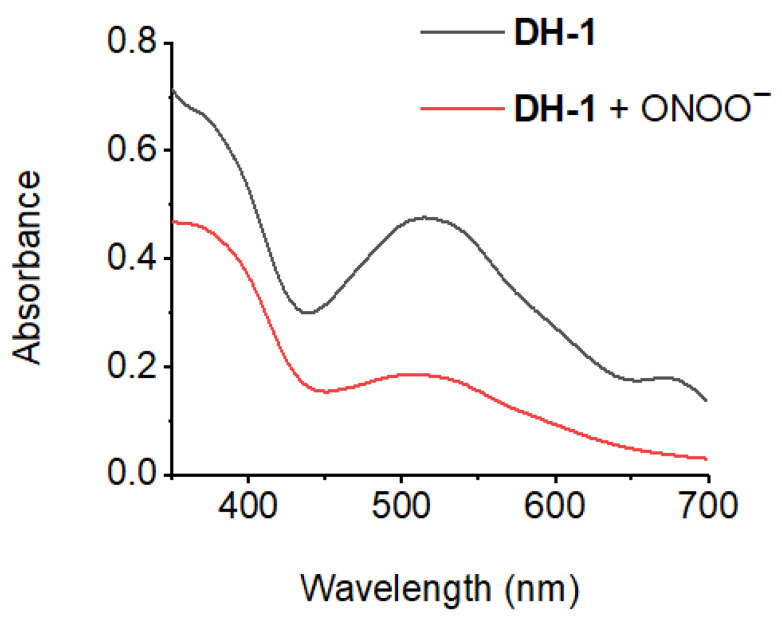
Absorption spectra of **DH-1** in the solution before (black) and after (red) the addition of ONOO^−^.

Initially, the spectroscopic properties of the probe **DH-1** were studied. It can be seen from Figure 1 that the probe showed a clear absorption peak at about 500 nm without ONOO^−^. After the addition of 4 equivalent (eq.) ONOO^−^, the absorbance of the probe was significantly reduced. Such absorption changes may be attributed to the reaction of probe **DH-1** with ONOO^−^, which caused the cleavage of the double bond by oxidation, and the destruction of the conjugated system. In addition, the proposed mechanism was further verified via MS, as shown in Figure S4. After reaction with ONOO^−^, probe **DH-1** indeed converted to **Rho-keto** with a mass peak at *m*/*z* = 390.1 [M]^+^ [12,13]. The results indicated that our probe design and sensing mechanism for ONOO^−^ detection was rational.

In addition, the excitation spectra of the probe before and after reaction with ONOO^−^ were investigated. As shown in Figure S5, the excitation intensity was almost undetectable, since the probe itself was non-fluorescent (quantum yield < 0.04). Once ONOO^−^ was added to the solution, the excitation intensity of the probe showed a maximum of approximately 400 nm. Therefore, we chose 400 nm as the excitation wavelength for the subsequent fluorescence property study.

**Figure 2:**
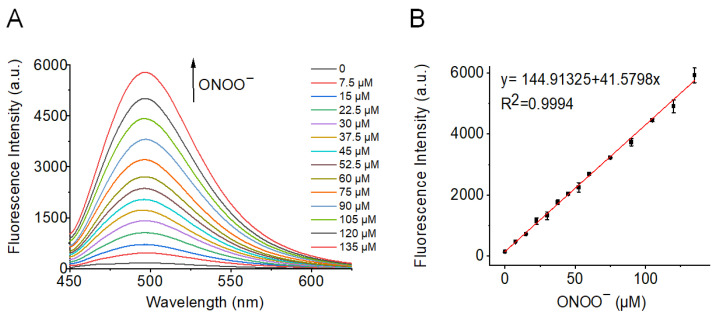
Fluorescence titration study. (**A**) Fluorescence spectra changes in **DH-1** after the addition of different concentrations of ONOO^−^. (**B**) Linear plot of the fluorescence emission of **DH-1** versus ONOO^−^. λ_ex_ = 400 nm; λ_em_ = 496 nm. The data were expressed as the mean ± standard deviation (SD) (n = 3).

We then explored the emission spectra of the probe upon the addition of various concentrations of ONOO^−^. The results shown in Figure 2 indicate that the fluorescence emission was remarkably enhanced, along with the titration of ONOO^−^. Moreover, there was a good linearity (R^2^ = 0.9994) between the fluorescence intensity of **DH-1** and the ONOO^−^ concentration (0~135 μM). Also, the detection limit was calculated to be 0.74 μM, which was lower than that of the previously reported ONOO^−^ probes listed in Table S1. This indicated that probe **DH-1** was rather sensitive to ONOO^−^, which would be favorable for intracellular ONOO^−^ detection with high sensitivity.

**Figure 3:**
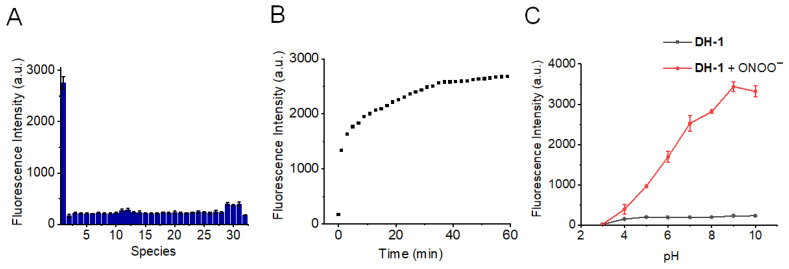
(**A**) Fluorescence responses of **DH-1** to various biologically relevant species. 1. ONOO^−^ (60 μM); 2. blank (i.e., probe only); 3. arginine (1 mM); 4. cysteine (1 mM); 5. glutamic acid (1 mM); 6. glutathione (1 mM); 7. homocysteine (1 mM); 8. histidine (1 mM); 9. serine (1 mM); 10. alanine (1 mM); 11. H_2_O_2_ (100 μM); 12. HOCl (100 μM); 13. Ag^+^ (100 μM); 14. Ca^2+^ (100 μM); 15. Fe^2+^ (100 μM); 16. K^+^ (100 μM); 17. Mg^2+^ (100 μM); 18. Na^+^ (100 μM); 19. Zn^2+^ (100 μM); 20. Al^3+^ (100 μM); 21. CO_3_^2−^ (100 μM); 22. NO_2_^−^ (100 μM); 23. NO_3_^−^ (100 μM); 24. S^2−^ (100 μM); 25. SO_3_^2−^ (100 μM); 26. S_2_O_3_^2−^ (100 μM); 27. SO_4_^2−^ (100 μM); 28. HSO_3_^−^ (100 μM); 29. *t*-BuOOH (100 μM); 30. •OH (100 μM); 31. NO (100 μM); 32. O_2_^•−^ (100 μM). (**B**) Dynamic fluorescence changes in **DH-1** after the addition of ONOO^−^ within 1 h. (**C**) pH effects on the fluorescence intensity of **DH-1** with (red) and without (black) ONOO^−^. λ_ex_ = 400 nm; λ_em_ = 496 nm. The data were expressed as the mean ± standard deviation (SD) (n = 3).

Since many species in the biological system may potentially affect the detection performance of the probe; therefore, we assessed the selectivity of the probe **DH-1**. In this experiment, various biological components (e.g., amino acids, anions, metal cations) were added to the probe solution to compare the fluorescence signal. It can be seen in Figure 3A that, except for ONOO^−^, the addition of analytes had little effect on the fluorescence emission, whereas ONOO^−^ could result in a significant fluorescence increase. It should be pointed out that reactive oxygen/nitrogen species, such as H_2_O_2_, HOCl, *t*-BuOOH, •OH, NO, as well as O_2_^•−^, did not cause significant fluorescence interference for ONOO^−^ detection. The response performance of **DH-1** will not be affected by reactive oxygen/nitrogen species. Thus, the probe **DH-1** showed high selectivity to ONOO^−^ in the complex biological samples.

We further investigated the fluorescence kinetic properties of the probe. From Figure 3B, it can be noticed that the fluorescence of **DH-1** increased rapidly in the presence of ONOO^−^. Also, the fluorescence signal reached its maximum in approximately 0.5 h. The time-course study indicated a rapid reaction of **DH-1** with ONOO^−^. In addition, considering that pH is a critical factor for molecular probes, we tested the fluorescence signal variations of **DH-1** within a pH range from 3 to 10. It can be noted in Figure 3C that the probe alone was almost unaffected by pH. After the addition of ONOO^−^, the fluorescence intensity of the probe **DH-1** enhanced greatly in terms of physiological pH, demonstrating its good responsiveness to ONOO^−^ in the detecting system. Moreover, we observed an interesting phenomenon: the fluorescence intensity of probe **DH-1** with ONOO^−^ in an acidic environment was lower than in a neutral or alkaline environment. The possible reaction mechanism of the probe with ONOO^−^ followed two steps. The first step was the nucleophilic addition of ONOO^−^, which attacked the C=C bond; the second step was the unstable intermediate transformation into the **Rho-keto** form. The alkaline environment was beneficial to the nucleophilic addition reaction, thereby causing higher fluorescence at an alkaline pH than at an acidic pH. The same phenomenon could also be found in other reported papers about ONOO^−^ probes [34,35].

### 2.3. Mitochondrial Targeting Ability of the Probe ***DH-1***

Prior to cellular imaging, we carried out an MTT test to explore the probe **DH-1**’s cytotoxicity. It can be noted in Figure S6 that cell survival was hardly influenced after the cells were incubated with various doses of **DH-1**. This indicated that **DH-1** had good biocompatibility.

**Figure 4:**
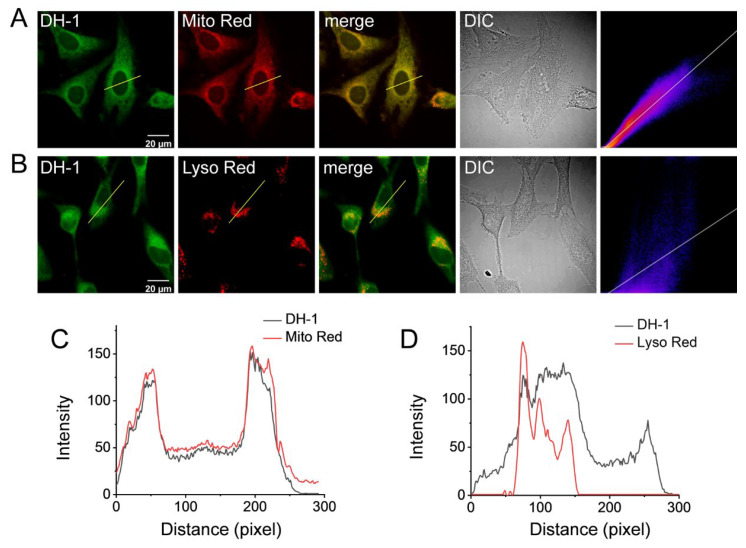
Co-localization fluorescence imaging of 5 μM **DH-1** and 100 nM Mito Red (**A**) or Lyso Red (**B**) in HeLa cells. (**C**) Intensity profiles within the ROI (region of interest; yellow line in the merge image in panel A). (**D**) Intensity profiles within the ROI (yellow line in the merge image in panel B). Pearson’s coefficient of Mito Red and **DH-1** was 0.97; Pearson’s coefficient of Lyso Red and **DH-1** was 0.37. **DH-1**: λ_ex_ = 405 nm, λ_em_ = 500–540 nm; Mito Red: λ_ex_ = 561 nm, λ_em_ = 570–620 nm; Lyso Red: λ_ex_ = 561 nm, λ_em_ = 570–620 nm.

**Figure 5:**
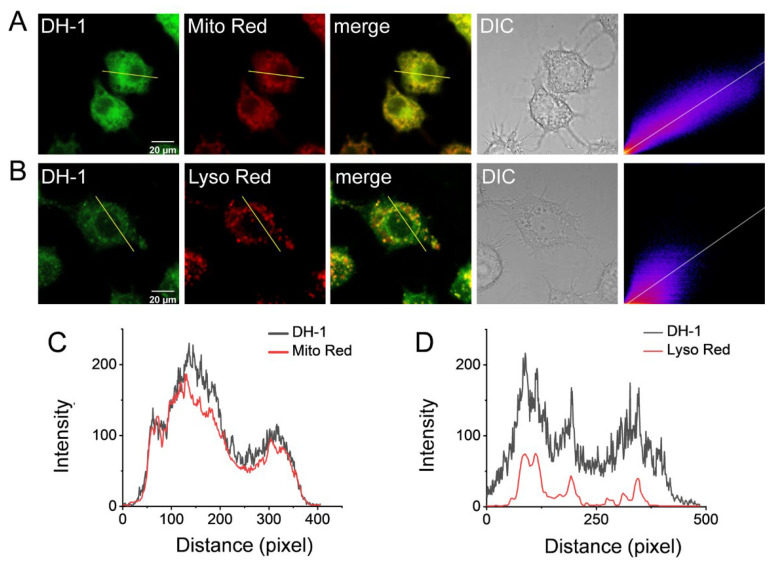
Co-localization fluorescence imaging of 5 μM **DH-1** and 100 nM Mito Red (**A**) or Lyso Red (**B**) in RAW264.7 cells. (**C**) Intensity profiles within the ROI (yellow line in the merge image in panel A). (**D**) Intensity profiles within the ROI (yellow line in the merge image in panel B). Pearson’s coefficient of Mito Red and **DH-1** was 0.92; Pearson’s coefficient of Lyso Red and **DH-1** was 0.41. **DH-1**: λ_ex_ = 405 nm, λ_em_ = 500–540 nm; Mito Red: λ_ex_ = 561 nm, λ_em_ = 570–620 nm; Lyso Red: λ_ex_ = 561 nm, λ_em_ = 570–620 nm.

For the purpose of probing **DH-1**’s ability to localize in different organelles, co-localization assays were carried out using the commercially available mitochondrial dye (Mito Red) or lysosomal dye (Lyso Red). Before the co-localization experiment, cells were incubated with the endogenous drugs lipopolysaccharide (LPS, 100 ng/mL) and murine recombinant interferon-γ (IFN-γ, 100 ng/mL) to generate endogenous ONOO^−^. It can be seen in Figure 4A,C that, in HeLa cells, the green fluorescence of **DH-1** overlapped well with the red fluorescence of Mito Red, and a rather high Pearson’s coefficient of 0.97 was determined. Meanwhile, we employed Lyso Red for comparison. It can be observed in Figure 4B,D that the overlap between **DH-1** and Lyso Red was small, whereas the corresponding Pearson’s coefficient was only 0.37. The results indicated that **DH-1** was able to effectively and selectively localize in mitochondria. Furthermore, we next conducted the co-localizing assay in RAW264.7 cells, and similar phenomena were observed, as shown in Figure 5. Hence, it could be concluded that **DH-1** was capable of targeting the mitochondria in cells.

### 2.4. Imaging of ONOO^−^ by the Probe ***DH-1*** in Cells

**Figure 6:**
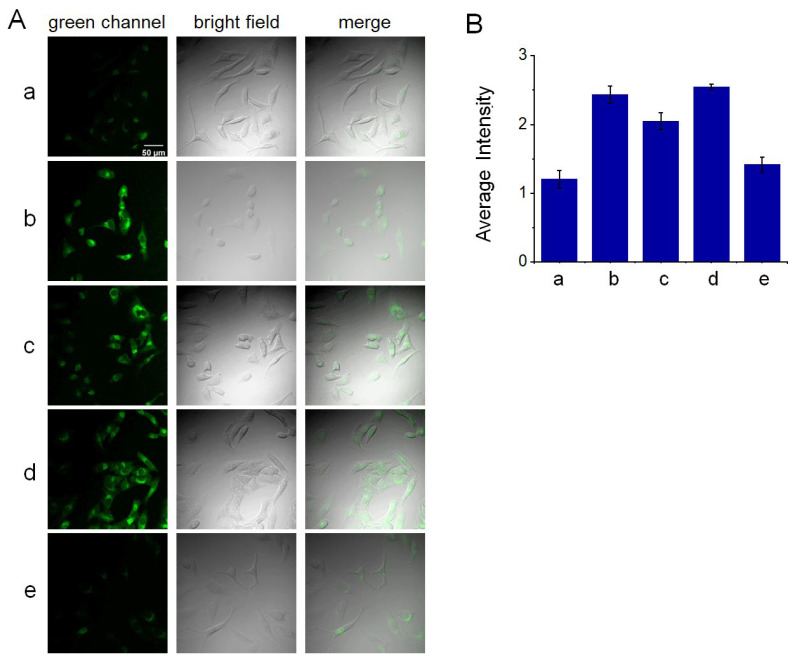
Cellular imaging of ONOO^−^ using **DH-1** in HeLa cells. (**A**) Confocal imaging of HeLa cells incubated at various conditions: (**a**) HeLa cells were incubated with 5 μM **DH-1** for 10 min. (**b**) HeLa cells were pretreated with 200 μM of 3-morpholinosydnonimine hydrochloride (SIN-1) for 2.5 h, then incubated with **DH-1**. (**c**) HeLa cells were pretreated with 100 ng/mL of LPS and 100 ng/mL of IFN-γ for 9 h, then incubated with **DH-1**. (**d**) HeLa cells were pretreated with LPS and IFN-γ for 9 h, then cultivated with 10 nM of PMA for 30 min, followed by incubation with **DH-1**. (**e**) HeLa cells were pretreated with LPS and IFN-γ for 9 h, then cultivated with 10 nM of PMA for 30 min, followed by incubation with 1 mM of AG for 30 min; finally, the cells were incubated with **DH-1**. (**B**) Cellular fluorescence intensity quantitation in panel A. All the cells in the field of vision were used for statistical quantitation, and the cell number was >10 in one separate experiment. The data were expressed as the mean ± standard deviation (SD) of three separate measurements.

**Figure 7:**
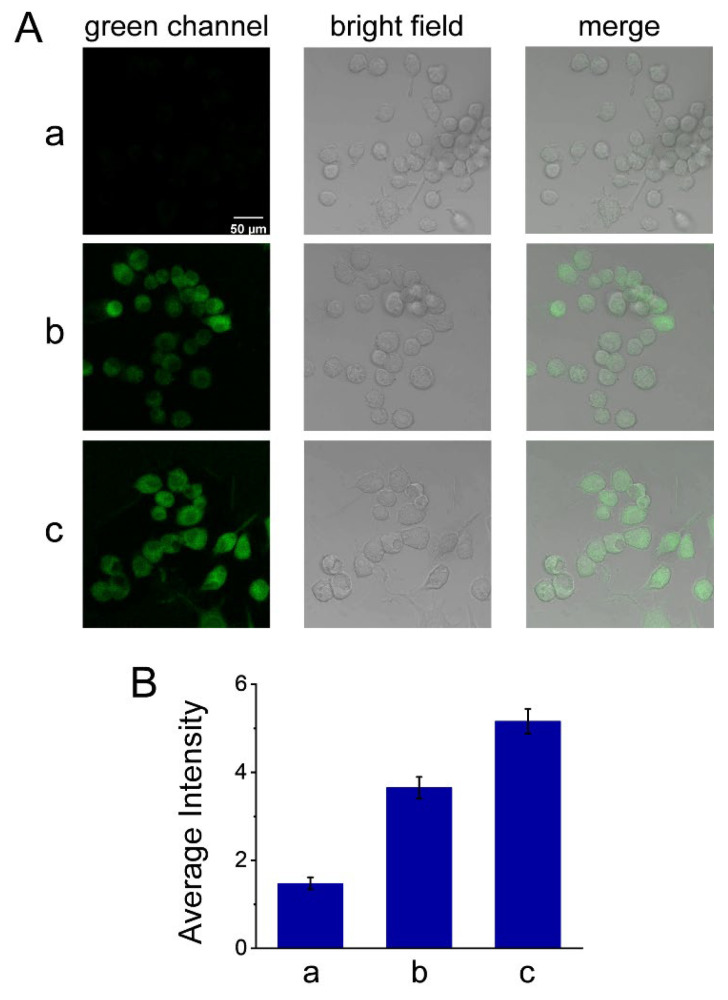
Cellular imaging of ONOO^−^ using **DH-1** in RAW264.7 cells. (**A**) Confocal imaging of RAW264.7 cells cultivated at various conditions: (**a**) RAW264.7 cells were incubated with 5 μM **DH-1** for 10 min. (**b**) RAW264.7 cells were first stimulated with 100 ng/mL of LPS and 100 ng/mL of IFN-γ for 10 h, followed by incubation with **DH-1**. (**c**) RAW264.7 cells were first stimulated with LPS and IFN-γ, and then cultivated with 10 nM of PMA for 30 min, followed by incubation with **DH-1**. (**B**) Cellular fluorescence intensity quantitation in panel A. All the cells in the field of vision were used for statistical quantitation, and the cell number was >10 in one separate experiment. The data were expressed as the mean ± standard deviation (SD) of three separate measurements.

Encouraged by the outstanding spectroscopic responses and specific mitochondria-targeting ability, we subsequently attempted to verify the reactivity of **DH-1** towards intracellular ONOO^−^. It can be seen in Figure 6A that when HeLa cells were cultivated with **DH-1** alone (control), we only observed very weak fluorescence. However, if cells were first stimulated with 200 μM of SIN-1, which was a kind of exogenous ONOO^−^ donor, the fluorescence became brighter. In addition, after treatment with the endogenous drugs LPS and IFN-γ, more pronounced fluorescence could be noted than that observed in control cells. Moreover, a subsequent incubation with phorbol-12-myristate-13-acetate (PMA) also produced strong fluorescence. Furthermore, in order to confirm that the increase in intracellular signal was ascribed to the production of endogenous ONOO^−^, a contrast group with aminoguanidine (AG, an inhibitor of nitric oxide synthase) was compared at the same time. It can be noted in image e in Figure 6A that the fluorescence intensity was remarkably diminished once the cells were incubated with LPS, IFN-γ, and PMA in the presence of AG. The above data well proved that **DH-1** was specifically responsive to intracellular ONOO^−^. The corresponding quantitative intensity statistics explicitly demonstrated these fluorescence changes (Figure 6B). The results suggested that the probe **DH-1** could track the intracellular ONOO^−^ fluctuations in response to the stimulation of exogenous and endogenous drugs. More importantly, these responses were not only observed in HeLa cells, but we also observed similar experiment phenomena in RAW264.7 cells (Figure 7), further indicating the desirable fluorescence responses of **DH-1** in living cells.

### 2.5. Fluorescence Imaging of ONOO^−^ during Ferroptosis

**Figure 8:**
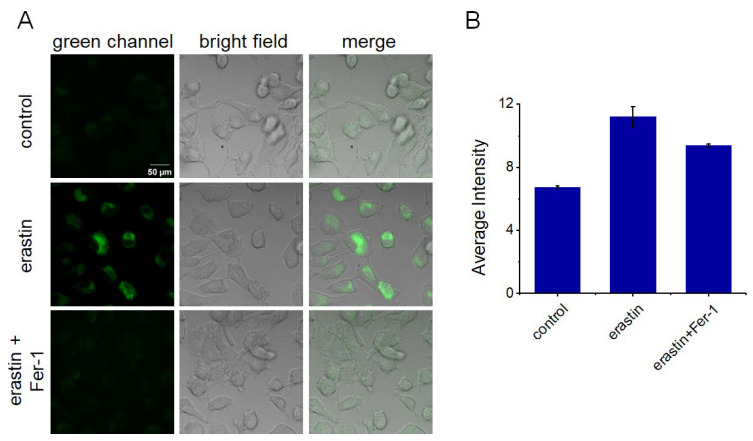
Cellular ONOO^−^ imaging during ferroptosis process in HeLa cells. (**A**) Confocal fluorescence images of cells incubated at various conditions. First row (control): cells were cultivated with **DH-1** (10 µM) for 30 min; second row: cells were cultivated with erastin (40 µM) for 15 h, and next treated with **DH-1**; third row: cells were cultivated with erastin (40 µM) for 15 h, and next treated with Fer-1 (10 µM) for 15 h, followed by incubation with **DH-1**. (**B**) Cellular fluorescence intensity quantitation in panel A. λ_ex_ = 405 nm; and λ_em_ = 500–540 nm. All the cells in the field of vision were used for statistical quantitation, and the cell number was >10 in one separate experiment. The data were expressed as the mean ± standard deviation (SD) of three separate measurements.

According to the previous literature, ferroptosis is one kind of cell death resulting from the iron-dependent accumulation of lipid peroxides associated with ROS. Among the ROS family, ONOO^−^ is related to lipid peroxidation and glutathione depletion during the ferroptosis process [45]. In this study, we explored the biological application of **DH-1** for detecting ONOO^−^ during ferroptosis. Herein, erastin, which is a common inducer of ferroptosis, was employed. HeLa cells were pretreated with erastin for 15 h and then incubated with **DH-1** for 30 min. It could be seen from Figure 8 that the fluorescence intensity was significantly enhanced compared with control cells without the stimulation of erastin. In addition, we carried out the inhibition experiment by using ferrostatin-1 (Fer-1), which is a common ferroptosis inhibitor. As displayed in the third row of Figure 8A, intracellular fluorescence intensity was largely weakened in the presence of Fer-1, which could further prove that the ONOO^−^ produced during the ferroptosis process indeed resulted in the fluorescence signal changes. The results showed that the probe **DH-1** may be a promising instrument for monitoring the dynamic ONOO^−^ changes in erastin-induced ferroptosis.

## 3. Materials and Methods

### 3.1. General

Nuclear magnetic resonance (NMR) spectra (including ^1^H and ^13^C NMR) were acquired using Bruker Avance-400 spectrometer. HRMS were obtained using Q-TOF6510 spectrograph (Agilent, Santa Clara, CA, USA). The pH of the solution was adjusted using a PHSJ-3F pH meter (LeiCi, Shanghai, China). Absorption spectra were collected using a U-3900 spectrophotometer (Hitachi, Tokyo, Japan). Emission spectra were measured using F-4600 spectrophotometer (Hitachi, Japan). Cellular fluorescence images were captured using FV3000 confocal scanning microscope (Olympus, Tokyo, Japan).

Ultrapure water was used in all the tests. All chemicals were provided by commercial sources and were of analytical grade. They were directly used without further purification. Mito Red and Lyso Red were purchased from KeyGEN BioTECH Co. Ltd., Nanjing, China.

### 3.2. Synthesis of the Probe ***DH-1***

The probe **DH-1** was prepared following the synthetic route outlined in Scheme 2, in which compound **Rho** was prepared using the previous method [46]. **Rho** (190 mg, 0.4 mmol) and ferrocenecarboxaldehyde (43 mg, 0.2 mmol) were added to the solution of toluene (10 mL) and n-butanol (10 mL). The mixture was stirred at 90 ºC for 4 h. Next, the solvent was evaporated. Subsequently, the residues were purified via silica gel chromatography with CH_2_Cl_2_/MeOH (15:1, *v*/*v*) as the eluent to provide **DH-1** (yield: 79%). ^1^H NMR (400 MHz, DMSO-*d*_6_) δ 7.94 (d, *J* = 7.6 Hz, 1H), 7.79 (t, *J* = 7.6 Hz, 1H), 7.67 (t, *J* = 7.6 Hz, 1H), 7.32 (d, *J* = 8.0 Hz, 1H), 7.06 (s, 1H), 6.52 (d, *J* = 2.4 Hz, 1H), 6.44 (dd, *J* = 9.2, 2.4 Hz, 1H), 6.37 (d, *J* = 8.8 Hz, 1H), 4.54 (s, 2H), 4.38 (s, 2H), 4.18 (s, 5H), 3.36 (q, *J* = 6.8 Hz, 4H), 2.73–2.83 (m, 1H), 2.52–2.60 (m, 1H), 1.75–1.85 (m, 1H), 1.57–1.70 (m, 2H), 1.47–1.56 (m, 1H), 1.10 (t, *J* = 6.8 Hz, 6H). ^13^C NMR (100 MHz, DMSO-*d*_6_) δ 152.25, 149.42, 147.21, 135.71, 130.31, 128.75, 127.34, 126.96, 125.12, 124.03, 123.94, 109.30, 106.03, 104.76, 97.33, 86.80, 81.35, 70.34, 70.27, 69.66, 69.54, 69.43, 44.15, 27.32, 23.10, 22.34, 12.85. HRMS *m*/*z*: calcd for C_35_H_34_FeNO_3_^+^ [M]^+^: *m*/*z* 572.1883; found: *m*/*z* 572.1879.

### 3.3. Spectroscopic Measurements

**DH-1** was dissolved in dimethyl sulfoxide to obtain stock solution (10^−3^ mol/L). A 5 mL sample of the mixture solution containing **DH-1** (final concentration, 15 μM) was combined with an appropriate volume of PBS and ONOO^−^ reacted at 37 °C for 1 h. Subsequently, 3 mL of the solution was transferred to the quartz cell to collect the absorbance and fluorescence spectra.

### 3.4. Culture of Cells

HeLa and RAW264.7 cells were cultured in DMEM, and the detailed culture conditions were the same as in the previous literature [47]. Cytotoxicity of probe **DH-1** was evaluated using standard MTT method.

### 3.5. Colocalization Experiments

RAW264.7 or HeLa cells were pretreated with LPS (100 ng/mL) and IFN-γ (100 ng/mL) for 9–10 h to generate endogenous ONOO^−^. Subsequently, cells seeded in glass-bottom dish were co-stained with **DH-1** (5 μM) and Mito Red (100 nM) or Lyso Red (100 nM) for 10 min at 37 °C. After being washed with PBS buffer 3 times, the cell images were captured using an Olympus FV3000 microscope. The excitation was set at 405 nm for **DH-1** or 561 nm for Mito Red and Lyso Red; the corresponding fluorescence emissions were collected at 500–540 nm for **DH-1** or 570–620 nm for Mito Red and Lyso Red.

### 3.6. Intracellular Fluorescence Imaging

Control (without stimulation): cells were incubated with **DH-1** (5 μM) for 10 min.

Exogenous drug stimulation: cells were incubated with the exogenous drug SIN-1 (200 μM) for 2.5 h, and then rinsed with PBS buffer, followed by incubation with **DH-1** (5 μM) for 10 min.

Endogenous drug stimulation: in the first group, cells were incubated with the endogenous drugs LPS (100 ng/mL) and IFN-γ (100 ng/mL) for 9–10 h, and then rinsed with PBS buffer, followed by incubation with **DH-1** (5 μM) for 10 min. In the second group, cells were first incubated with LPS and IFN-γ, and then treated with PMA (10 nM) for 0.5 h, followed by incubation with **DH-1** for 10 min. In the third group, cells were first incubated with LPS and IFN-γ, and then treated with PMA (10 nM) for 0.5 h, followed by incubation with AG (1 mM) for 0.5 h; finally, the cells were incubated with **DH-1** for 10 min.

Finally, all the groups were subjected to cell fluorescence imaging with 405 nm excitation, and the corresponding fluorescence emissions were collected from 500 to 540 nm.

## 4. Conclusions

In conclusion, we have developed a new molecular fluorescence probe, **DH-1,** to detect cellular ONOO^−^ based on the rhodamine skeleton. **DH-1** showed many desirable properties, including high sensitivity, unique specificity, and physiological pH response, along with good biocompatibility. Moreover, **DH-1** was capable of targeting mitochondria and detecting intracellular exogenous and endogenous ONOO^−^ changes. In addition, we have successfully applied **DH-1** for ONOO^−^ imaging in the ferroptosis process; intracellular fluorescence could be efficiently attenuated in the presence of a ferroptosis inhibitor. Thus, the probe might act as an effective molecular tool for probing the biological functions of ONOO^−^ in clinical practice.

## Data Availability

The data presented in this study are available in the article and Supplementary Materials.

## Supplementary Materials

The following supporting information can be downloaded at: https://www.mdpi.com/article/10.3390/molecules28247976/s1, Figure S1: ^1^H NMR spectrum of **DH-1**; Figure S2: ^13^C NMR spectrum of **DH-1**; Figure S3: HRMS of **DH-1**; Figure S4: MS of the reaction solution containing **DH-1** and ONOO^−^; Figure S5: Excitation spectra of **DH-1** before and after reaction with ONOO^−^; Figure S6: Viability of HeLa cells treated with **DH-1** at different concentrations (0–20 μM); Table S1: Comparison of **DH-1** with other ONOO^−^ fluorescent probes. References [48,49,50,51,52].
