# Supplementary material for: A Small-Molecule Fluorescent Probe for the Detection of Mitochondrial Peroxynitrite
Source: Molecules. 2023 Dec 6;28(24):7976. doi: 10.3390/molecules28247976 (PMC10745935; doi:10.3390/molecules28247976)
Supplement: Supplementary file 1 [file molecules-28-07976-s001.zip › molecules-2627527-supplementary.pdf]

Supplementary Materials

A Small-Molecule Fluorescent Probe for the Detection of Mitochondrial Peroxynitrite

Han Dong, Meng-Yu Tang, Shili Shen *, Xiao-Qun Cao and Xiao-Fan Zhang *

Institute of Optical Functional Materials for Biomedical Imaging,
School of Chemistry and Pharmaceutical Engineering,
Shandong First Medical University & Shandong Academy of Medical Sciences,
Taian 271016, China; xqcao@sdfmu.edu.cn (X.-Q.C.)
* Correspondence: slshen@sdfmu.edu.cn (S.S.); zhangxiaofan0912@163.com (X.-F.Z.)

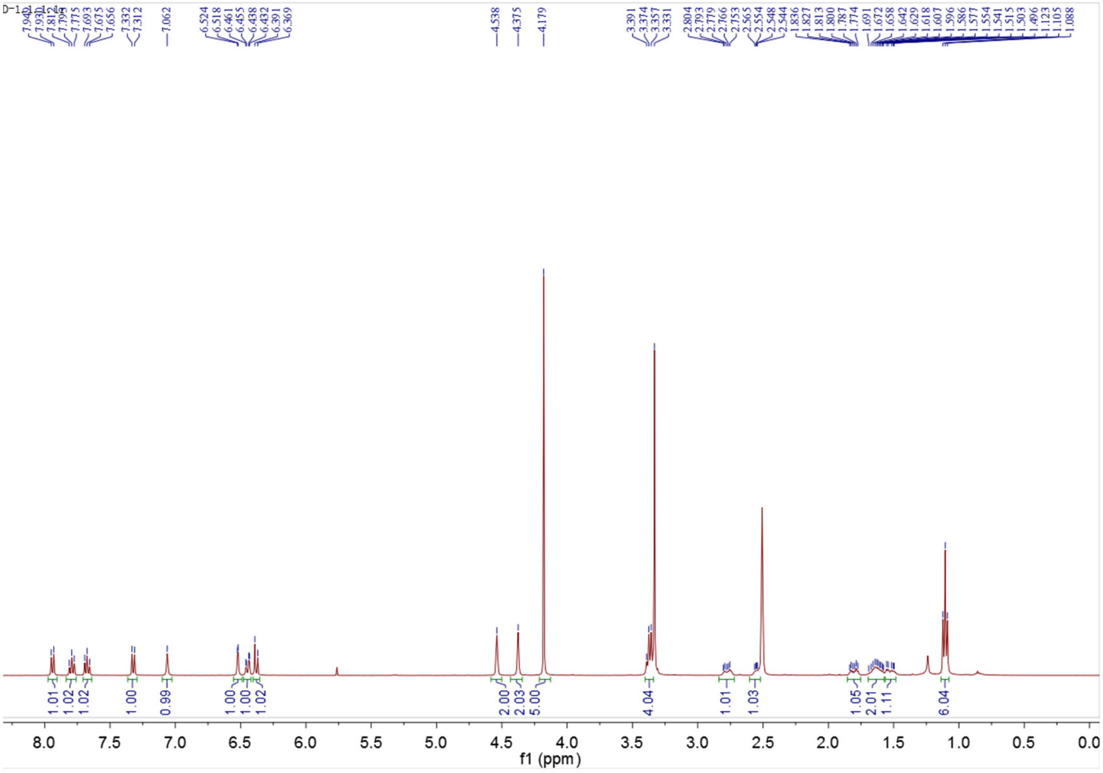

Figure S1. ^{1}H NMR spectrum of **DH-1** in DMSO-d_6.

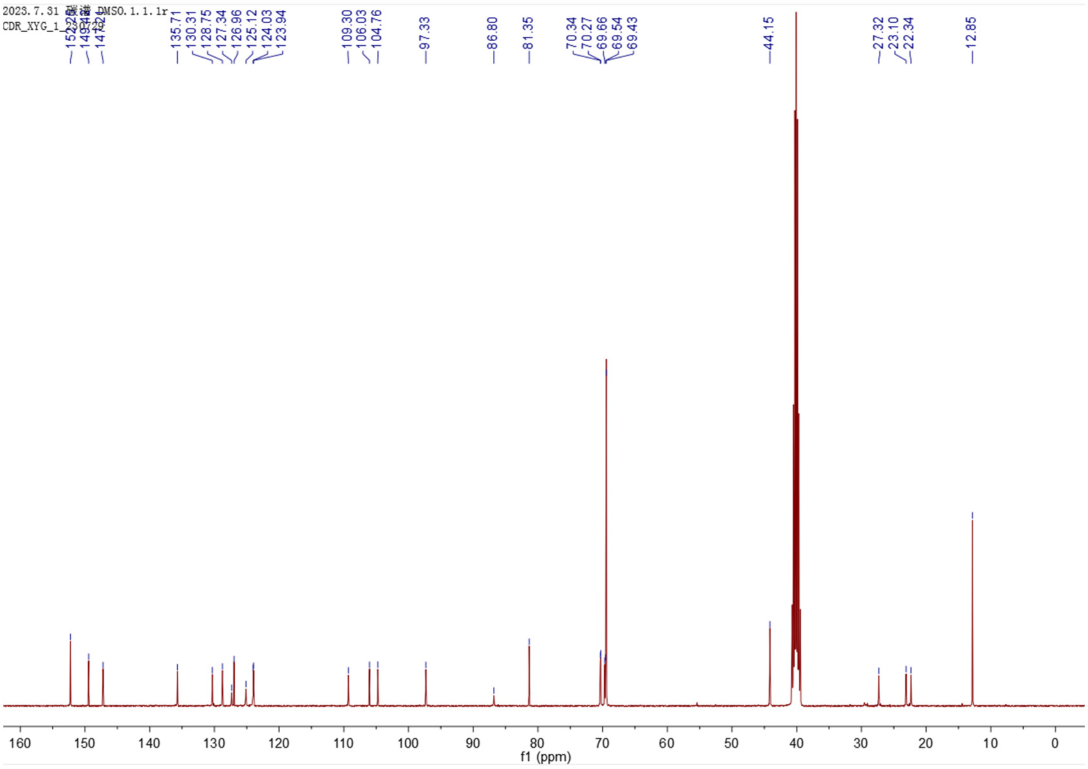

Figure S2. ^{13}C NMR spectrum of **DH-1** in DMSO-d_6.

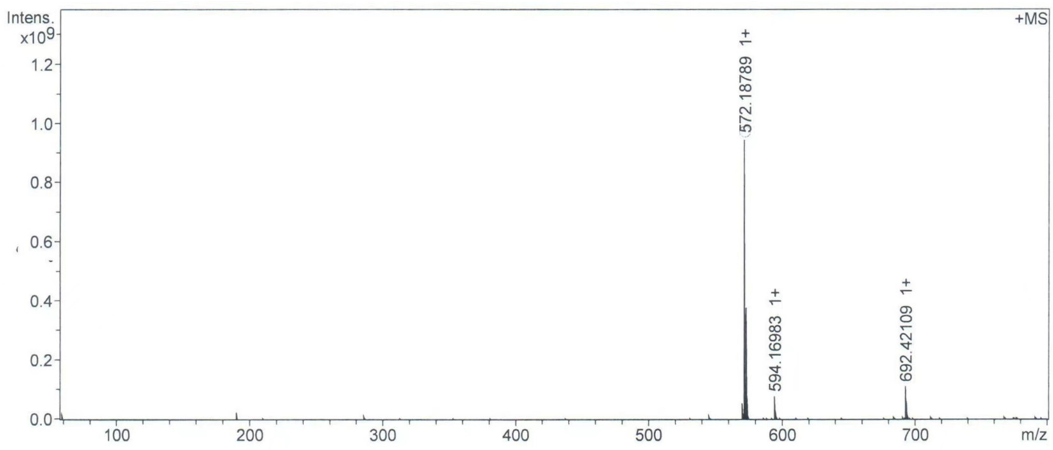

Figure S3. HRMS of **DH-1**.

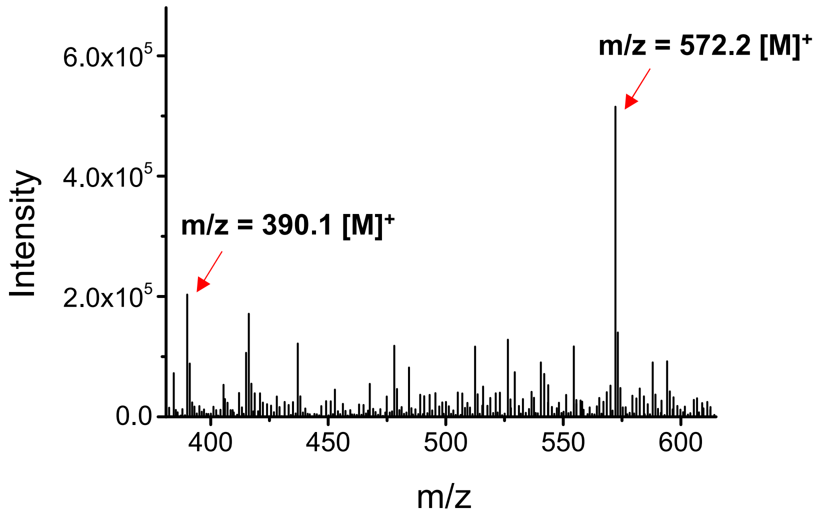

Figure S4. MS of the reaction solution containing **DH-1** and $ONOO^-$.

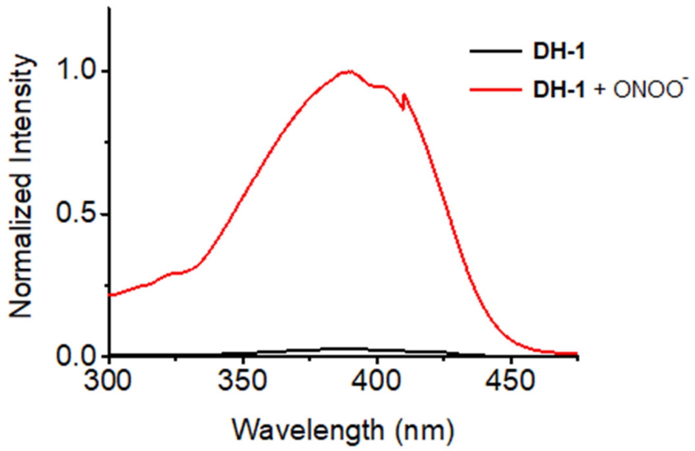

Figure S5. Excitation spectra of **DH-1** before and after reaction with $ONOO^-$.

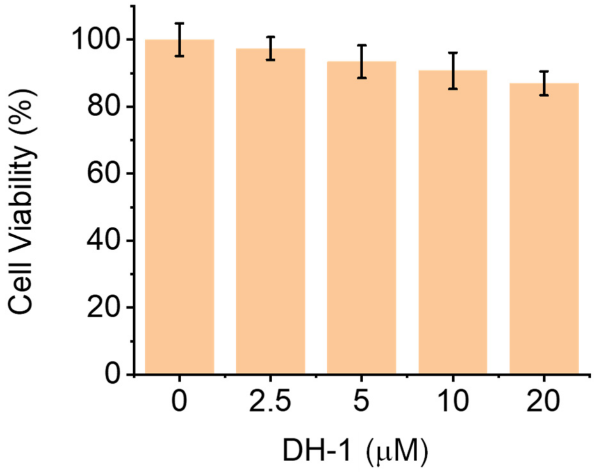

Figure S6. Viability of HeLa cells treated with **DH-1** at different concentrations (0-20 μM). The cell viability without probe was defined as 100%. Results were the mean ± standard deviation of five separate measurements.

Table S1. Comparison of **DH-1** with other $ONOO^-$ fluorescent probes.

Ref.	[1]	[2]	[3]	[4]	[5]	**This work**
Detection limit (μM)	0.93	100	2.5	0.917	1.8	**0.74**

References

[1] Liu, Y.Y.; Ma, Y.Y.; Lin, W.Y. Construction of a bi-functional ratiometric fluorescent probe for detection of endoplasmic reticulum viscosity and $ONOO^-$ in cells and zebrafish. *Sens. Actuators B Chem.* **2022**, *373,* 132742.

[2] Song, Z.; Mao, D.; Sung, S.H.P.; Kwok, R.T.K.; Lam, J.W.Y.; Kong, D.; Ding, D.; Tang, B.Z. Activatable fluorescent nanoprobe with aggregation-induced emission characteristics for selective in vivo imaging of elevated peroxynitrite generation. *Adv. Mater*. **2016**, *28,* 7249-7256.

[3] Kim, J.; Park, J.; Lee, H.; Choi, Y.; Kim, Y. A boronate-based fluorescent probe for the selective detection of cellular peroxynitrite. *Chem. Commun.* **2014,** *50*, 9353-9356.

[4] Yu, F.B.; Li, P.; Wang, B.S.; Han, K.L. Reversible near-infrared fluorescent probe introducing tellurium to mimetic glutathione peroxidase for monitoring the redox cycles between peroxynitrite and glutathione in vivo. *J. Am. Chem. Soc.* **2013**, *135,* 7674-7680.

[5] Wu, J.; Yang, Y.Z.; Zhang, L.; Wang, H.; Yang, M.; Yuan, J.L. A visible-light-excited Eu^{3+} complex-based luminescent probe for highly sensitive time-gated luminescence imaging detection of intracellular peroxynitrite. *J. Mater. Chem. B* **2017**, *5,* 2322-2329.
